# Reproductive characteristics and thyroidal function in relation with season in Khuzestan buffalo (*Bubalus bubalis*) bulls

**Published:** 2014

**Authors:** Sadegh Mayahi, Morteza Mamouei, Saleh Tabatabaei, Khalil Mirzadeh

**Affiliations:** *Department of Animal Sciences, Faculty of Animal and Food Sciences, Ramin Agriculture and Natural Resources University, Mollasani, Ahvaz, Iran*

**Keywords:** Buffalo, Season, Semen quality, Thyroid hormones

## Abstract

High ambient temperature is the major constraint on Buffalo productivity. The aim of this study was to evaluate the reproductive performance and thyroid gland function in winter and summer seasons in Khuzestan buffalo bulls. Six male indigenous buffaloes of Khuzestan with nearly the same age (2-3 years old) and weight were used. Semen and blood samples through jugular vein were collected, every two weeks throughout the summer and winter seasons. The thyroid hormones and thyrotropin stimulating hormone (TSH) concentration in blood serum were measured by radioimmunoassay method. Semen quality was determined, using computer assisted sperm analyzer (CASA) and routine methods. The concentration of thyroxin (T_4_) was lower in winter than summer (*p* ≤ 0.05). The level of T_3 _uptake was higher in cold season than that of in hot season (*p* ≤ 0.05). The differences of tri-iodotyronine (T_3_) and TSH concentrations, as well as free thyroxin index were not significant between seasons. The semen volume and spermatozoa parameters including concentration, progressive motility, linear velocity, mean velocity, beat cross frequency, linear coefficient and straightness coefficient were higher in winter than summer (*p* ≤ 0.05). Semen pH and amplitude of lateral head displacement of spermatozoa were higher in summer than winter (*p *≤ 0.05). In winter, there was positive correlation between spermatozoa concentration and T_3_ value of blood serum (*p* ≤ 0.05). There were positive correlations between values of semen volume and T_4_, progressive spermatozoa motility percent and TSH, as well as, total motility of spermatozoa and TSH in summer (*p* ≤ 0.05). In general, thyroid function and semen quality of Khuzestan buffaloes may be affected by seasons.

## Introduction

The domestic buffalo is an indispensable livestock resource to millions of smallholders in developing countries, particularly in Asia. Buffalo is very sensitive to high ambient temperature and direct exposure to the sun. This is due to the fact that buffalo bodies absorb a great deal of solar radiation because of their dark skin and sparse coat or hair. In addition to that they possess a less efficient evaporative cooling system due to their rather poor sweating ability. ^1^ Under most smallholder production systems, the reproductive efficiency of buffalo is determined by many factors including climate, manage-ment, nutrition and diseases.

In past years, selection of male buffaloes based on reproductive performance is mentioned. Therefore, a lot of research has been done on sexual performance and semen quality of various breeds of buffalo in different parts of the world.^2^^,^^3^ Concentrations of thyroid hormones, thyroxine (T_4_) and tri-iodotyronine (T_3_), in cattle were influenced by a variety of environmental factors, including ambient temperature and dietary components and intake.^4^ Under hot weather conditions in the Khuzestan province in Iran (Maximum ambient temperature in summer is about 49 ˚C), exposure to high environmental temperature is an important factor which limits the animal production.

Thyroid gland is one of the most sensitive organs to the ambient heat variation.^5^ It was reported that thermal exposure acts directly on the hypothalamic pituitary axis and causes a reduction in thyrotropin stimulating hormone (TSH) and therefore, thyroidal hormone secrations.^6^^,^^7^ There are some evidences to indicate that the factors that have inhibitory effect on thyroid function could reduce reproductive efficiency.^8^

The modulation of thyroid hormone is the major trends of growth and the general metabolism. Seasonal changes in thyroid activity have been shown in different animals. Thyroid gland plays the most important role in metabolism, growth and reproduction activity in the body. The metabolic hormone, T_4_, has been implicated in the physiological regulation of energy balance as well as in maintaining normal reproductive function in mammals.^9^ Thyroid gland has been suggested to have a significant effect on the male reproductive tract, spermatogenesis, and male fertility. ^10^ Thyroid hormone acts directly on Sertoli cells to inhibit the proliferation, while stimulate their differentiation and maturation. Thyroid hormone is the best described modulator of Sertoli cells differentiation.^11^ Thyroid is a metabolically important gland, so that has suggested to being essential for the normal maintenance of reproductive function as its impairment may inhibit reproductive system. The role of T_3_ in Sertoli cell development is incomplete yet, however, T_3 _appears to exert pleiotropic effects on Sertoli cell maturation.^12^ The administration of T_3_ into the brain caused about similar morphological changes in testicular growth under short-day conditions.^13^^,^^14^ In sheep, thyroid hormones are known to be involved in both changes in the responsiveness of the gonadotropin releasing hormone (GnRH) axis to estrogen-negative feedback at the transition to anestrus, and in the steroid-independent cycles in LH pulse frequency. Thyroid hormones are also known to have essential roles in development and plasticity of the central nervous system, and seasonal plasticity in the GnRH system has also been reported.^15^ Follicle-stimulating hormone (FSH) and luteinizing hormone (LH) hormones are produced by the anterior pituitary. The production of these two hormones is stimulated by GnRH secreted by hypothalamus. It is largely known, how LH, FSH and testosterone hormones regulate spermatogenesis.The effect of transient induction of neonatal hypo- and hyperthyroidism on post-natal testis development, Sertoli cell proliferation and different spermatogonial cell types has been investigated.^16^^, ^^17^ The concept that thyroid gland is not that important in adult testicular physiology and male fertility, is now being questioned. Alterations in the gonadotropins in response to change in the thyroid hormone levels indicate fine coordination between hypothalamus-pituitary-thyroid (HPT) and hypothalamus-pituitary-gonadal (HPG) axes. In farm animals, iodine deficiency is the most important health problems in the world. Iran is an endemic area of iodine deficiency. Iodine deficiency may be due to iodine deficiency in thyroid gland or secondarily in association with consuming the goitrogenic materials. Thiocyanate is one of the goitrogenic agents that inhibits iodine uptake by the thyroid gland and can interfere with the binding of iodine to the thyroid hormone. The weight of thyroid gland in buffalo is about 18 ± 2 g and the secretion of two hormones, T_3_ and T_4_ in various physiological phenomena is applied.^18^ The aim of this study was to evaluate the reproductive and thyroid gland activity between winter and summer seasons in Khuzestan buffalo bulls.

## Materials and Methods

Six sexually mature Khuzestan buffalo (*Bubalus bubalis*) of 2-3 years of age with similar nutrition and nearly the same weight (230 ± 20 kg) were used. Blood samples (from jugular vein) and semen were collected every 2 weeks throughout the summer (average ambient temperature 37 ˚C, max. 49 ˚C, min. 25 ˚C) and winter (average 13 ˚C, max. 25 ˚C, min. 3 ˚C) seasons for the period of six months. After centrifuging the blood samples at 3000 rpm for 5 min, serum samples were placed in to microtubes and stored in –22 ˚C until hormonal analysis. The concentrations of T_4_, total T_3_, TSH and T_3 _uptake were determined by radioimmunoassay (RIA) method. Semen from all the experimental buffaloes were collected using an artificial vagina and the volume, mass activity, motility percentage, pH and sperm concentration were recorded. Semen volume was recorded directly from graduated test tubes. The pH of semen was measured with a paperturnsole.^19^ Sperm motility was assessed by computer assisted sperm analyzer (CASA; Sperm Vision 3.0, Minitube, Ingersoll, Canada) before being diluted in semen extender. Spermatozoa motion parameters were curvilinear velocity (VCL; μm sec^-1^), linear velocity (VSL; μm sec^-1^), mean velocity (VAP; μm sec^-1^), straightness coefficient (STR, %), linear coefficient (LIN, %), frequency of head displacement or beat cross frequency (BCF, Hz) and amplitude of lateral head displacement (ALH, µm). ^20^^, ^^21^

The differences of semen and thyroidal values between winter and summer seasons were analyzed by paired-sample *t*-test in SPSS (Version 16; SPSS Inc., Chicago, USA). Pearson's correlation coefficient test was used for the evaluation the correlations between subjecting parameters. Data are presented as mean ± SE.

## Results

The concentration of T_4_ in summer was significantly higher than winter (*p* ≤ 0.05); while, the value of T_3 _uptake in winter was higher than summer (*p* ≤ 0.05). The amounts of T_3_, TSH and free thyroxin index did not differ through hot and cold seasons (Table 1). As shown in Tables 2 and 3, the semen volume and spermatozoa parameters, namely the concentration, progressive motility, VSL, VAP, BCF, LIN and STR were higher in winter when compared with summer (*p* ≤ 0.05). However, semen pH and ALH of spermatozoa were higher in summer than winter (*p *≤ 0.05). There were not significant differences for total motility percentage and VCL of spermatozoa between summer and winter seasons. In winter, there was significantly positive correlation between spermatozoa concentration and T_3_ value of blood serum (*p* ≤ 0.05, r = 0.84), (Table 4). As it is clear in Table 4, there were positive correlations between values of semen volume and T_4_ (*p* ≤ 0.05, r = 0.84), progressive spermatozoa motility percentage and TSH (*p* ≤ 0.05, r = 0.88), as well as the total motility of spermatozoa and TSH in summer season (*p* ≤ 0.05, r = 0.86).

**Table 1:** Blood serum thyroidal hormone values in cold and warm seasons in Khuzestan buffalo bulls

	**Tri-iodotyronine ** **(ng m** **L** ^-1^ **)**	**Thyroxin** **(mg dL** ^-1^ **)**	**Thyrotropin stimulating hormone ** **(ng mL** ^-1^ **)**	**Tri-iodotyronine ** **uptake** ** (%)**	**Free thyroxin index**
**Winter** **Summer**	2.05 ± 0.10 2.07 ± 0.22	4.16 ± 0.68^b^ 5.08 ± 0.74^a^	0.03 ± 0.01 0.06 ± 0.01	34.30 ± 0.18^a^ 30.89 ± 0.48^b^	141.95 ± 23.14 155.72 ± 21.53

a, b Different superscripts in the same column indicate significant difference at *p *< 0.05.

**Table 2:** Semen quality values in cold and warm seasons in Khuzestan buffalo bulls

	**Semen volume** **(** **mL** **)**	**Sperm concentration** **(×10** ^6^ **mL**^-1^**)**	**Progressive sperm** **motility** ** (%)**	**Total motility of** **sperm** ** (%)**	**Semen** ** pH**
**Winter** **Summer**	5.01 ± 0.28^a^ 3.40 ± 0.25^b^	289.87 ± 10.25^a^ 244.91 ± 11.93^b^	84.09 ± 3.20^a^ 78.98 ± 2.67^b^	92.61 ± 0.88 91.89 ± 2.08	6.30 ± 0.04^a^ 7.13 ± 0.06^b^

a, b Different superscripts in the same column indicate significant difference at *p *< 0.05.

**Table 3:** Spermatozoa motility characteristics in cold and warm seasons in Khuzestan buffalo bulls

	**VCL** ** (** **μm** ** sec** ^-1^ **)**	**VSL (** **μm** ** sec** ^-1^ **)**	**VAP (** **μm** ** sec** ^-1^ **)**	**ALH (** **µm** **)**	**BCF (** **Hz** **)**	**LIN** ** (%)**	**STR** ** (%)**
**Winter** **Summer**	47.59 ± 1.15 43.69 ± 2.02	29.01 ± 1.32^a^ 21.56 ± 0.62^b^	35.96 ± 1.16^a^ 28.61 ± 0.95^b^	1.08 ± 0.06^b^ 2.65 ± 0.10^a^	5.11 ± 0.27^a^ 4.67 ± 0.19^b^	56.42 ± 3.89^a^ 45.77 ± 1.56^b^	71.02 ± 2.85^a^ 65.69 ± 1.68^b^

VCL: curvilinear velocity; VSL: linear velocity; VAP: Mean velocity; ALH: Amplitude of lateral head displacement; BCF: Frequency of head displacement or beat cross frequency; LIN: Linear coefficient; STR: Straightness coefficient.

a, b Different superscripts in the same column indicate significant difference at *p *< 0.05.

**Table 4:** Correlation between semen quality and blood serum thyroidal hormones of Khuzestan buffalo in winter and summer seasons. Numbers are correlation coefficients (r); ^*^ indicate significant difference at* p *< 0.05.

	**Tri-iodotyronine**		**Thyroxin**		**Thyrotropin stimulating hormone**	
	**Winter**	**Summer**	**Winter**	**Summer**	**Winter**	**Summer**
**Semen** **volume** **Semen** **pH** **Progressive** **sperm** **motility** **(%)** **Sperm** **concentration (×10**^6^ **mL**^-1^**)** **Total motility of sperm (%)**	0.16 –0.30 0.21 0.84٭ 0.45	0.30 0.30 0.52 –0.68 0.41	0.15 –0.37 0.18 0.66 0.73	0.80٭ 0.10 0.44 –0.53 0.15	0.29 0.52 0.27 –0.67 –0.08	0.20 0.40 0.88٭ 0.10 0.86٭

## Discussion

In this study, there were significant differences for semen quality parameters of buffaloes between winter and summer seasons. Weather significantly influenced most of the motion characteristics of buffalo bull spermatozoa. Progressive sperm motility in the winter was significantly higher than summer season. This result is consistent with the findings of Gamboa *et al.* in horses and Gundogan in sheep.^22^^, ^^23^ However, it was in contrast with results of Lessard*et al.* in North American wild buffalo.^20 ^This mismatched results could be attributed to differences in weather conditions of the study area, different livestock breeds, feeding, age and management conditions. Seasonal variation in semen quality and fertility of bulls has been reported in both temperate and tropical areas. The significant highly seasonal effect was found in the Brahman (*Bos indicus*) bulls for both the clinical reproductive parameters and spermiogram tests.^24^ In the study of Chacon *et al*., a significant variation was observed in the mean percentage of overall sperm motility during one year period, which decreased significantly from 63.00% in September to 47.00% in December.^24^ In our study, the VAP, VSL, LIN, BCF and STR of spermatozoa had a significant increase in winter than in summer. However, ALH was the higher in summer. This finding is in agreement with the observations in swamp buffalo,^25^ and is in contrast with results in the Murah buffaloes.^26^ This could be due to differences in breed and environmental factors.^25^ The present study showed that semen volume was significantly higher in winter when compared with summer. This finding is consistent with the reports of Koonjaenak in swamp buffalo.^25^ The pH of semen in the present study was significantly lower in winter than summer. This observation was in consistent with findings of Temwong *et al*. in swamp buffalo.^22^^,^^27^ The findings of this study showed that the acidification rate of semen was significantly lower in winter than summer which can be associated with higher spermatozoa concentration and their metabolism in winter season. Also, acidification of semen in winter can represent the high number of live sperms. In our results, sperm concentration was significantly higher in winter compared to summer. This outcome was consistent with the findings in swamp buffalo.^25^ However, it was in contrast with results of Khan*et al.* in Nili-Ravi buffalo.^28^ These variations are due to the breed and the climate under which that breed have been reared. Intensity or mildness of the season itself and the other factors such as feed and water availability and the level of the management standards are also contributing factors. Generally, extreme weather conditions are not favorable for producing good quality semen.^28^ There were significantly positive correlations between TSH level and sperm motility as well as semen volume and concentration of thyroxine in the summer and between T_3 _value and sperm concentration in the winter. Similar observations were reported by other study in buffalo.^29^ It seems that thyroid hormones can affect the testis via two pathways; a direct effect on testis, and an effect on gonadotropin. Thyroid hormone receptor protein is minimal in adult testis and is expressed at high levels in the developing testis.^30^ Thyroid hormones have also been reported to be essential for changes in GnRH neurosecretory systems that are fundamental to reproductive seasonality.^30^ It seems likely that thyroid hormone could exert indirect effects on structural changes in testicular activity and its consequences for qualitative and quantitative changes in sexual function and semen characteristics of Khuzestan buffalo. The T_3_ is an important factor in regulating the development of neonatal testis. It is involved in both Sertoli cell proliferation and differentiation.^31^ Studies on the effect of thyroid hormones directly on the testes have indicated that there is a minimal change in oxygen consumption when T_4_ is present in testicular slice incubations.^32^ Overall, the results of this study showed a significant difference in reproductive performance of male buffaloes in winter and summer seasons.

Therefore, although the mating of male Khuzestan buffalo can be done throughout the year, reproductive performance and fertile potential of animal can be affected by seasonal variations. The changing pattern of sex hormone secretion and metabolism of thyroid hormones in the winter and summer can play a significant role in the qualitative and quantitative changes in semen characteristics and reproductive performance of males. 

It is concluded that semen quality and blood serum T_3 _uptake value of Khuzestan buffalo bulls was significantly higher in cold season than warm season. Whereas, the concentration of T_4 _was higher in summer when compared to winter. There was significantly positive correlation between spermatozoa concentration and blood serum T_3 _level in cold season. In summer, there were positive correlations between semen volume and T_4 _value, progressive spermatozoa motility percent and TSH level, as well as, the total motility percent of spermatozoa and TSH amount.
